# Near-ultraviolet photon-counting dual-comb spectroscopy

**DOI:** 10.1038/s41586-024-07094-9

**Published:** 2024-03-06

**Authors:** Bingxin Xu, Zaijun Chen, Theodor W. Hänsch, Nathalie Picqué

**Affiliations:** 1 Max-Planck Institute of Quantum Optics, Garching, Germany; 2 Faculty of Physics, Ludwig-Maximilian University of Munich, Munich, Germany; 3 Max-Born Institute for Nonlinear Optics and Short-Pulse Spectroscopy, Berlin, Germany; 4 Present Address: Ming Hsieh Department of Electrical and Computer Engineering, University of Southern California, Los Angeles, CA USA

**Keywords:** Optical spectroscopy, Electronic structure of atoms and molecules, Optical metrology, Optical spectroscopy, Frequency combs

## Abstract

Ultraviolet spectroscopy provides unique insights into the structure of matter with applications ranging from fundamental tests to photochemistry in the Earth’s atmosphere and astronomical observations from space telescopes^1–8^. At longer wavelengths, dual-comb spectroscopy, using two interfering laser frequency combs, has become a powerful technique capable of simultaneously providing a broad spectral range and very high resolution^9^. Here we demonstrate a photon-counting approach that can extend the unique advantages of this method into ultraviolet regions where nonlinear frequency conversion tends to be very inefficient. Our spectrometer, based on two frequency combs with slightly different repetition frequencies, provides a wide-span, high-resolution frequency calibration within the accuracy of an atomic clock, and overall consistency of the spectra. We demonstrate a signal-to-noise ratio at the quantum limit and an optimal use of the measurement time, provided by the multiplexed recording of all spectral data on a single photon-counter^10^. Our initial experiments are performed in the near-ultraviolet and in the visible spectral ranges with alkali-atom vapour, with a power per comb line as low as a femtowatt. This crucial step towards precision broadband spectroscopy at short wavelengths paves the way for extreme-ultraviolet dual-comb spectroscopy, and, more generally, opens up a new realm of applications for photon-level diagnostics, as encountered, for example, when driving single atoms or molecules.

## Abstract (web summary)

We demonstrate a photon-counting approach that extends the unique advantages of spectroscopy with interfering frequency combs into regions where nonlinear frequency conversion tends to be very inefficient, providing a step towards precision broadband spectroscopy at short wavelengths and extreme-ultraviolet dual-comb spectroscopy.

## Main

Ultraviolet spectroscopy plays a pivotal role in studying electronic transitions in atoms and rovibronic transitions in molecules, essential for tests of fundamental physics and of quantum-electrodynamics theory^1,2^, determination of fundamental constants^3^, precision measurements^4^, optical clocks^5^, high-resolution spectroscopy supporting atmospheric chemistry^6^ and astrophysics^7,8^, as well as strong-field physics^11^.

The emerging dual-comb technique^9,12–23^ offers spectroscopy a unique host of features, which would provide a unique tool for vacuum- and extreme-ultraviolet spectroscopy. Dual-comb spectroscopy leverages frequency combs, spectra of evenly spaced phase-coherent laser lines that have revolutionized time and frequency metrology^24,25^. Dual-comb spectra span over a broad spectral bandwidth and their frequency scale may be directly calibrated within the accuracy of an atomic clock, whereas, as the transitions are interrogated with narrow laser lines, the well-defined instrumental line shape usually has a negligible contribution compared to that of the atomic or molecular profiles^9^. Dual-comb spectroscopy is also a complex technique that involves recording, over long durations, the time-domain interference between the two frequency combs with slightly different repetition frequencies. Dual-comb spectroscopy at present attracts tremendous interest in the infrared range.

Considerable progress has been achieved for generating frequency combs at short wavelengths and using them with techniques of narrow-band spectroscopy^1–5^. So far, high-resolution dual-comb spectroscopy with ultraviolet radiation has not been reported. Previous studies have demonstrated nonlinear spectroscopy in rubidium using two-photon excitation with combs at 384 THz (780 nm)^26,27^. Linear-absorption spectroscopy is of substantial interest in atmospheric science, and two proposals have discussed possible implementations based on high-power laser systems for reaching short wavelengths^28,29^. At present, several research groups are pursuing different approaches for experimental implementation^30–33^.

Here, we take a significant step towards precise spectroscopy over broad spectral bandwidths in the extreme-ultraviolet spectral region by demonstrating a new approach to dual-comb spectroscopy that is suitable for extremely low light levels, as encountered in frequency-comb photonics at short wavelengths. By using photon-counting technology, we have achieved precise, high-resolution, quantum-noise-limited near-ultraviolet dual-comb spectroscopy that operates at photon fluxes more than 10^6^ times lower than commonly used levels in dual-comb spectroscopy and other techniques of comb-based Fourier transform spectroscopy^9^. We demonstrate comb-line-resolved, high-resolution dual-comb absorption spectroscopy in the near-ultraviolet region. We achieve resolutions of 500 MHz (resolving power 1.5 × 10^6^) and 200 MHz (resolving power 4 × 10^6^) at the centre frequency of 772 THz (388 nm). Our innovative approach based on photon counting enables a quantum-noise-limited signal-to-noise ratio, particularly difficult to achieve in dual-comb spectroscopy. Our robust method for interferometry at low light levels overcomes the challenges posed by the low efficiency of nonlinear frequency conversion and, therefore, it lays a solid foundation for extending dual-comb spectroscopy to even shorter wavelengths.

## Principle of photon-counting dual-comb spectroscopy

We counterintuitively conduct dual-comb spectroscopy under extremely low light conditions, where, on average, fewer than one detector count occurs every 20 repetition periods of the comb. It is unlikely that two photons, one from each comb generator, would be present in the interferometer at the same time: Two different quantum paths interfere at the counter and the sum of their probability amplitudes provides the probability amplitude of detecting a count^10^. We operate with optical powers that are more than a million times weaker than those commonly used in dual-comb spectroscopy. In ref. ^10^, we had proposed the conceptual possibility of dual-comb spectroscopy in the photon-counting regime, however, its practical applicability has remained elusive, due to poor spectral resolution.

**Fig. 1:**
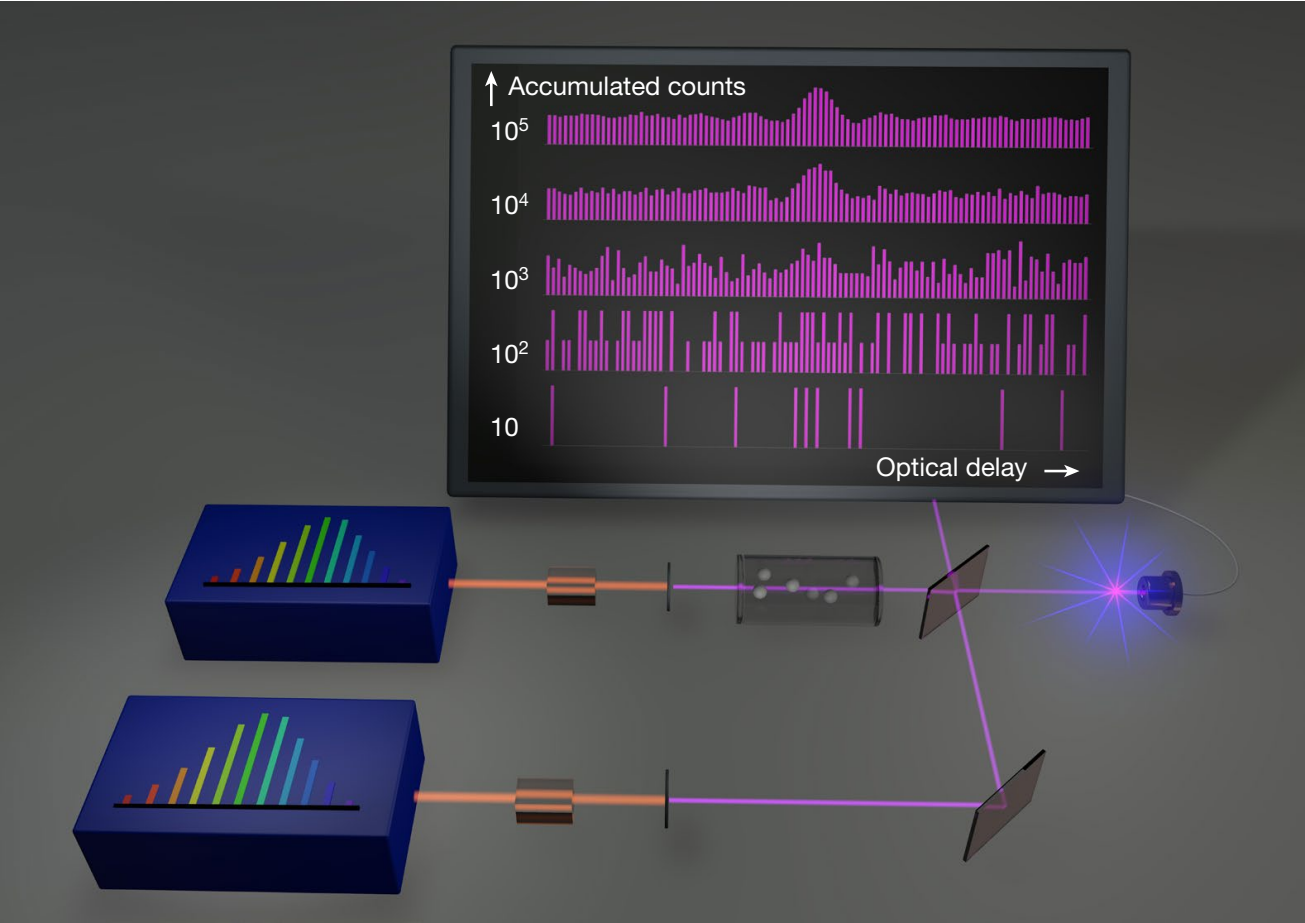
Principle of ultraviolet dual-comb spectroscopy with photon counting. The beam of a very low-light-level comb generator based on nonlinear frequency conversion of a near-infrared comb passes through an absorbing sample. It is superimposed on a beam splitter with the beam of another very low-light-level comb generator of slightly different pulse repetition frequency. One beam-splitter output is counted by a photon-counting detector. Fewer than one detector count occurs every 20 comb pulses. At power levels more than 10^6^ times weaker than usually used in dual-comb spectroscopy, the statistics of the detected counts carry the spectral information about the sample.

Two frequency-comb generators emit, at very low light levels, pulse trains with slightly different repetition frequencies, *f*_rep_ and *f*_rep_ + δ*f*_rep_, respectively (Fig. 1). The two beams of the two combs are combined on a beam splitter and their time-domain interference is measured on a fast photon counter. We record the detector counts as a function of time during a specific duration that we call a scan. A scan is initiated by a trigger signal generated in the instrument. Subsequent trigger signals repeat the same scans. By interferometrically controlling the relative timing and phase fluctuations between two combs, the time scale is made to correspond to a scale of optical delay between pairs of pulses. The detector counts at a given optical delay are added to the already accumulated counts of the same delay. Owing to the low light level, it is essential to accumulate many identical scans to reconstruct the interferograms with sufficient statistical data from photon counts. The sequence is repeated until the desired signal-to-noise ratio is achieved in the interferogram (Fig. 1 and Supplementary Video 1). Direct accumulation is only feasible if precise reproducibility of the interferometric scans is achieved. We achieve this by using well-controlled frequency combs and choosing experimental parameters that result in reproducible interferometric waveforms. In particular, we set δ*f*_ceo_ = 0 (modulo δ*f*_rep_), where δ*f*_ceo_ is the difference between the carrier-offset frequencies of the two combs. The sampling rate of the time bins should be an integer multiple of the difference in repetition frequencies δ*f*_rep._ Here, we demonstrate accumulation times of more than 1 hour with quantum-noise limited sensitivity.

Once the acquisition is completed with sufficient statistics, the interferogram can be processed similarly to dual-comb interferograms obtained at higher power. In the frequency domain, pairs of optical comb lines, one from each comb, produce radio-frequency beat notes on the detector, forming, in the radio-frequency domain, a frequency comb of line spacing δ*f*_rep_ and with a carrier-envelope offset frequency equal to zero. Optical frequencies are thus down-converted into radio frequencies, *m*δ*f*_rep_, where *m* is an integer.

## Near-ultraviolet spectra with electro-optic combs

**Extended Data Fig. 1:**
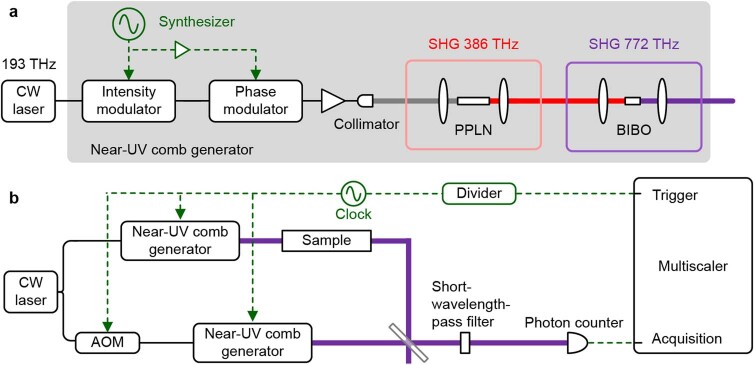
Detailed experimental setup for near-ultraviolet photon-counting dual-comb spectroscopy. **a,** A near-infrared continuous-wave laser is modulated by electro-optic modulators and frequency-doubled twice to generate a weak comb at 772 THz with an average power of a few 10^−11^ W. **b,** Two combs are generated as sketched in **a** from a single continuous-wave laser and are combined on a beam-splitter. One output of the beamsplitter is counted by a photon counter and accumulated as a function of arrival time after the trigger by a multi-scaler. Typically, a few 10^7^ counts per second are recorded, which corresponds to less than a count per comb repetition-frequency occurrence.

We illustrate the potential of near-ultraviolet dual-comb spectroscopy by using nonlinear frequency conversion of near-infrared electro-optic frequency combs (Fig. 1, Extended Data Fig. 1 and Methods). The electro-optic system, with its poor conversion efficiency in the ultraviolet, is well suited to test our approach to photon counting. Two frequency combs with slightly different repetition frequencies are generated from a continuous-wave laser that is intensity- and phase-modulated by electro-optic modulators at a centre frequency of 193 THz (1,550 nm). An acousto-optic modulator offsets the centre frequency of one comb, to measure the dual-comb spectrum without aliasing. The near-infrared combs are sequentially frequency doubled twice, once in a periodically poled lithium-niobate crystal and once in a BiB_3_O_6_ (BIBO) crystal. The near-ultraviolet combs of about 100 lines have a centre frequency tuneable between 770 and 774 THz, and a repetition frequency freely selectable between 100 kHz and 40 GHz. The two low-power ultraviolet frequency-comb beams are combined on a beam splitter. One output of the beam splitter is detected by a photon counter. The detected photon rate of the combined beam of the two combs is at most 5 × 10^7^ photons s^−1^. This corresponds to an average optical power per comb incident on the counter of about 50 pW, more than 1 million-fold weaker than that commonly used in dual-comb spectroscopy. The fringe visibility is on the order of 30% (Extended Data Table 1). The counts are sampled by a multiscaler at a rate ranging between 12.5 GHz and 500 MHz. The trigger signal for the data acquisition by the multiscaler is generated by frequency division of the 10 MHz clock signal.

**Fig. 2:**
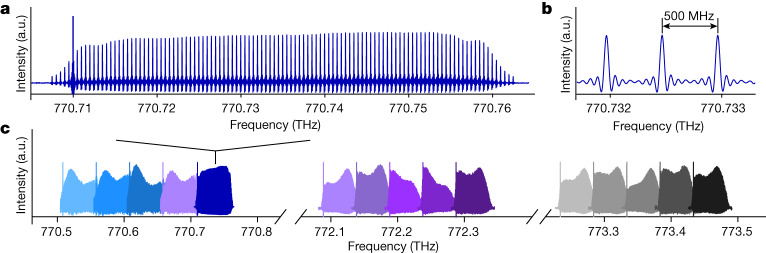
Near-ultraviolet photon-level dual-comb experimental spectra with resolved comb lines. **a**, Spectrum recorded at a detected rate of 2.7 × 10^7^ photons s^−1^ with about 100 comb lines. The *y* scale is linear. **b**, Magnified portion of **a** showing three comb lines with cardinal-sine line shapes. **c**, Illustration of the frequency agility: a sequential acquisition of 15 spectra spanning overall a bandwidth broader than 3 THz. a.u., arbitrary units.

**Extended Data Fig. 2:**
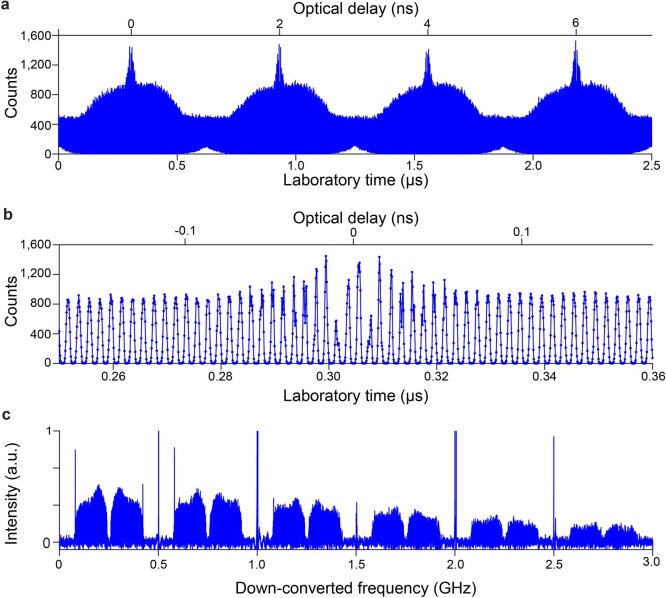
Photon-counting near-ultraviolet interferogram and spectrum at a sampling rate of 12.5 × 10^9^ bins per second. The count rate is 5 × 10^7^ counts s^−1^ and the accumulation time is 0.2 s. **a,** The interferogram features δ*f*_rep_ = 1.6 MHz and a trigger rate of 200 kHz. **b,** Magnified portion of **a** in the region of zero optical delay. **c,** The Fourier transform of the interferogram reveals the multiple aliases of the spectrum (12 in the representation) with decreasing intensity as the detection bandwidth drops.

The temporal build-up of an interferogram by accumulation of many scans is shown in Supplementary Video 1. An experimental dual-comb interferogram sampled at a rate of 12.5 GHz (Extended Data Fig. 2) recurs with a period of 625 ns, owing to a difference in repetition frequency δ*f*_rep_ of 1.6 MHz. A Fourier transform reveals the dual-comb spectrum, as in traditional dual-comb spectroscopy. A dual-comb spectrum centred at 770.73 THz contains more than 100 comb lines with a flat-top intensity distribution and results from an accumulation time of 255.5 s (Fig. 2a). The spectral resolution is equal to the line-spacing *f*_rep_ of 500 MHz. The span is 50 GHz. The instrumental line shape is a cardinal sine, as expected in an unapodized spectrum (Fig. 2b). By tuning the centre frequency of the continuous-wave near-infrared laser, a sequence of dual-comb spectra corresponding to 15 acquisitions centred at different frequencies is acquired over a total span of about 3 THz (Fig. 2c).

**Fig. 3:**
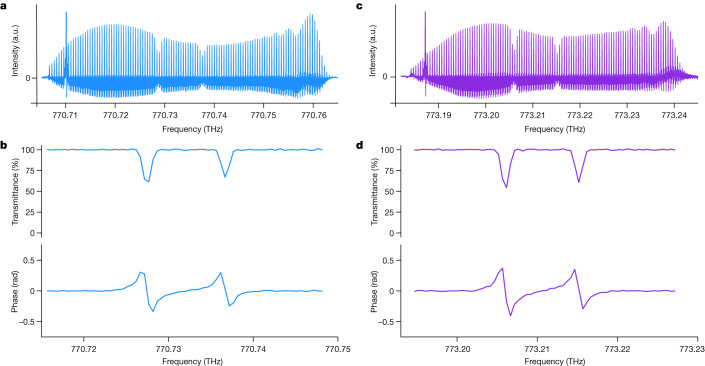
Photon-counting near-ultraviolet amplitude and phase spectra of weak transitions in ^133^Cs at a total average optical power of 90 pW and 500 MHz resolution. The *y* scale is linear. The spectra span over 50 GHz. **a**,**b**, 6S_1/2_–8P_1/2_ resonances. **a**, Amplitude spectrum with resolved comb lines. **b**, Transmittance and dispersion spectra, obtained by sampling the complex spectrum at the comb line positions, with a signal-to-noise ratio of 210. **c**,**d**, 6S_1/2_–8P_3/2_ resonances. **c**, Amplitude spectrum. **d**, Transmittance and dispersion spectra with a signal-to-noise ratio of 195.

**Extended Data Fig. 3:**
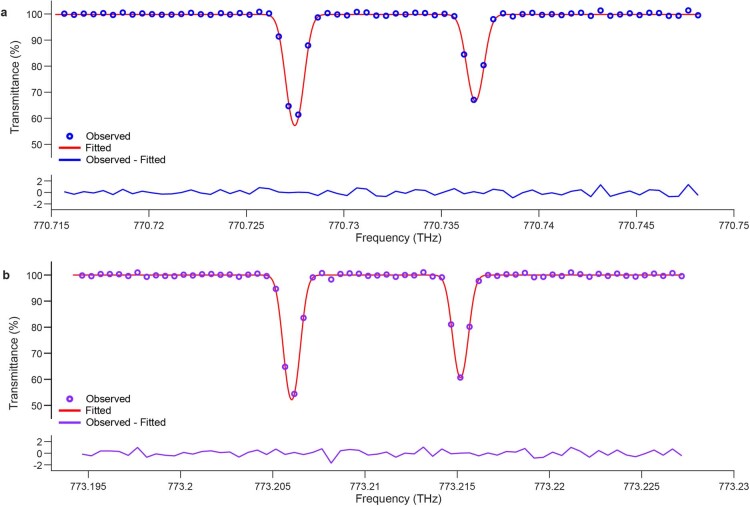
Line-position measurements in photon-counting near-ultraviolet spectra of ^133^Cs for the 6S_1/2_(*F* = 3,4)-8P_1/2_ and 6S_1/2_(*F* = 3,4)-8P_3/2_ transitions. **a,b**, 6S_1/2_(*F* = 3,4)-8P_1/2_ (**a**) and 6S_1/2_(*F* = 3,4)-8P_3/2_ (**b**) transitions. Doppler profiles (red) are adjusted to the experimental spectra (blue/violet dots). The transmittance scale is only plotted down to 50%. The differences ‘Observed-Fitted’ (solid blue/violet line) are plotted on a magnified scale. They do not show any systematic deviation, and their standard deviation is 0.5% in both spectra. The result of the fit also confirms that the instrumental line-shape can be neglected in the adjustment procedure.

**Extended Data Fig. 4:**
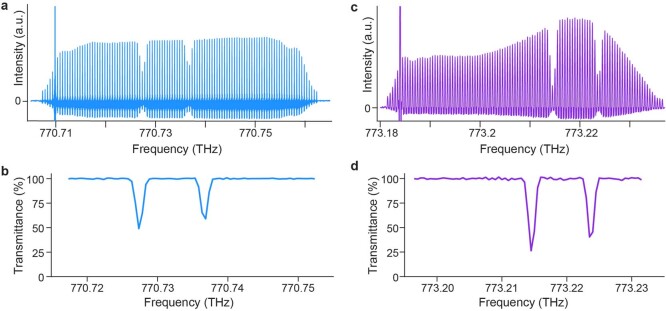
Photon-counting near-ultraviolet spectra of weak transitions in caesium when the two combs interrogate the absorption cell. The total average optical power of 60 pW and 500-MHz resolution. The y-scale is linear. The spectra span over 50 GHz. Because the two combs are attenuated by the resonances, the absorption profiles appear deeper than in Fig. 3. The spectral lineshapes are a convolution of the absorption experienced by the comb lines at different frequencies, thus the inversion of the spectra may not be as precise as in the configuration where only one comb interacts with the sample. **a,b,** 6S_1/2_-8P_1/2_ resonances with a signal-to-noise ratio of the transmittance baseline of 345. The count rate is 2.98 × 10^7^ s^−1^. The accumulation time is 270.4 s. **c,d,** 6S_1/2_-8P_3/2_ transitions with a signal-to-noise ratio of 205. The count rate is 2.83 × 10^7^ s^−1^. The accumulation time is 82.4 s.

Next, we illustrate the potential of our photon-counting dual-comb interferometer for absorption spectroscopy of the weak 6S–8P transitions in atomic caesium vapour. One of the comb beams passes through a heated caesium cell before the two comb beams are combined on a beam splitter. The interferograms are detected at a rate of 4.5 × 10^7^ counts s^−1^, which corresponds to an average optical power per comb of 45 × 10^−12^ W incident onto the detector of a quantum efficiency of 25% (Extended Data Table 1 and Methods). With roughly 100 comb lines, the power per comb line is estimated to be 4 × 10^−13^ W, simply calculated as the optical power divided by the number of comb lines. Spectra with resolved comb lines show the 6S_1/2_–8P_1/2_ and 6S_1/2_–8P_3/2_ transitions at 770.73 and 773.21 THz, respectively (Fig. 3a,c, respectively)^34^. The transmittance spectrum and the dispersion spectrum of the 6S_1/2_–8P_1/2_ transitions of a Doppler full-width at half-maximum of 1 GHz result from an accumulation time of 152 s (Fig. 3b). The two resonances are due to the hyperfine splitting in the 6S_1/2_ ground state (*F* = 3,4)_,_ while the hyperfine structure in the excited state is not resolved due to the Doppler broadening. The signal-to-noise ratio, determined as the inverse standard deviation of the normalized absorption baseline of the amplitude spectrum, is 210. The stronger 6S_1/2_–8P_3/2_ transitions are measured at a shorter accumulation time (64 s) at a signal-to-noise ratio of 195 (Fig. 3d). Absolute frequency measurements of the position of the strongest lines reach an uncertainty of 6 × 10^−9^, dominated by the statistical uncertainty (Methods, Extended Data Fig. 3 and Extended Data Table 2). When the two comb beams pass through the cell, only the transmittance spectrum is revealed (Extended Data Fig. 4).

**Extended Data Fig. 5:**
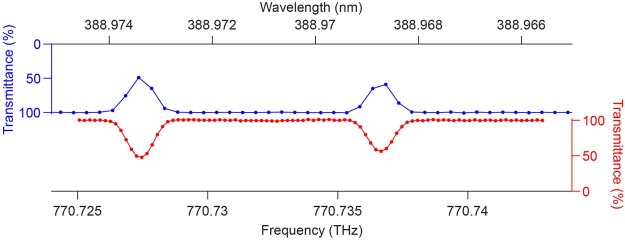
Repetition-frequency agility in photon-counting dual-comb spectroscopy. Experimental transmission spectra of the caesium 6S_1/2_- 8P_1/2_ transitions at a temperature of 378 K with a resolution of 500 MHz (blue, also in Extended Data Fig. 4a, b) and of 200 MHz (red). The resolution is freely chosen by adjusting the comb line spacing and resolving the comb lines in the spectra. The spectrum at 200-MHz comb line spacing results from a count rate of 2.3 × 10^7^ counts s^−1^, corresponding to an average power of 23 pW per comb incident on the detector. The accumulation time is 328 s.

One may argue that each spectrum does not span over extended regions. This feature of dual-comb spectroscopy with electro-optic modulators has been discussed in the context of infrared spectroscopy^20^: the moderate spectral span is compensated by the frequency agility of the continuous-wave laser and of the driving synthesizers: the centre frequency and the repetition frequency of the combs can be adjusted by a simple knob, and the small number of comb lines provides a high signal-to-noise ratio in short measurement times, with selectable spectral resolution. This makes the setup attractive for some experimental configurations such as atomic spectroscopy in which the spectra are not too dense and crowded. As an illustration of the adjustable resolution, the transmittance spectrum of the 6S_1/2_–8P_1/2_ transition at a resolution of 500 MHz is compared with that at a resolution of 200 MHz (Extended Data Fig. 5).

**Fig. 4:**
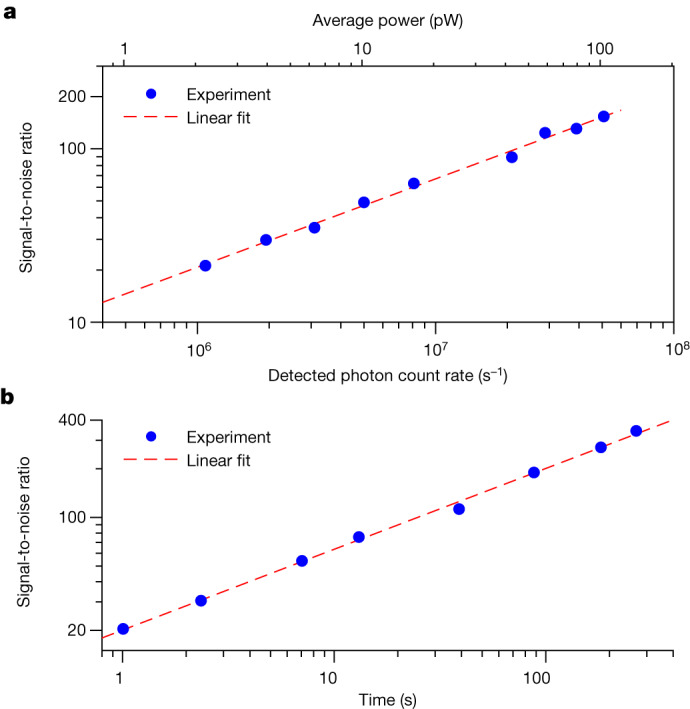
Quantum-noise-limited signal-to-noise ratio in photon-counting near-ultraviolet dual-comb spectroscopy. **a**, The signal-to-noise ratio of the transmittance baseline scales as the square root of the photon rate, showing that the quantum-noise limit is reached. The slope in the linear fit is 0.51 (±0.01). **b**, The signal-to-noise ratio also scales as the square root of the accumulation time, showing that mutual coherence of the two combs is maintained. The slope in the linear fit is 0.50 (±0.01).

Reducing technical noise sources to reach the quantum-noise limit is not easy in dual-comb spectroscopy. Most experiments have been limited by detector noise or intensity noise of the laser sources. In the experimental conditions of Extended Data Fig. 4a,b, we measure the experimental signal-to-noise ratio of the absorption baseline in the spectra as a function of count rate (Fig. 4a) and accumulation time (Fig. 4b). The experimental signal-to-noise ratio scales as the square root of the detected photon rate (Fig. 4a), which demonstrates that the signal-to-noise ratio is limited by counting statistics (also called quantum-noise limit or shot-noise limit). The experimental signal-to-noise is also in good agreement with a simple model of the quantum-noise-limited signal-to-noise ratio (Methods and Extended Data Table 1). The experimental signal-to-noise also scales with the square root of the accumulation time, showing that the interferometric coherence is maintained (Fig. 4b).

## Visible spectra with fibre lasers

Vacuum- and extreme-ultraviolet combs have only been generated as harmonics of near-infrared femtosecond mode-locked lasers^35^, so it is crucial to establish the suitability of such lasers for photon-counting dual-comb spectroscopy. We illustrate that, even with fibre mode-locked lasers attenuated to only ten million counts per second, the spectral information in amplitude and phase, of an absorbing sample can be precisely retrieved from the photon-counting statistics.

**Extended Data Fig. 6:**
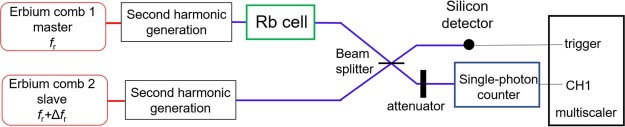
Experimental setup of visible photon-level dual-comb spectroscopy. Two frequency combs based on frequency doubled fiber lasers form a dual-comb interferometer at 384 THz. The beam of one comb interrogates a rubidium vapour cell. One output of the interferometer is strongly attenuated to an average power of 3 × 10^−12^ W, corresponding to one count every twenty-four laser pulses on average. The photon counts are counted by a photon-counter and a multiscaler accumulates them as a function of optical delay.

A pair of frequency-doubled erbium-doped femtosecond mode-locked fibre lasers generate two trains of optical pulses at a central frequency of 384 THz (Extended Data Fig. 6 and Methods). Their repetition frequencies are 100 MHz and their difference δ*f*_rep_ is equal to −12.5 kHz. One fibre comb is self-referenced against a radio-frequency clock. A feed-forward dual-comb technique^36^ establishes mutual coherence between the two frequency combs, effectively stabilizing their relative phase and timing fluctuations. The phase-matching properties of the periodically poled lithium-niobate crystals limit the spectral span to roughly 0.12 THz. The self-referenced comb passes through a Rb vapour cell, after which it is combined on a beam splitter with the second comb. At one of the beam-splitter outputs, the beam is attenuated to a detected count rate of 8.4 × 10^6^ counts s^−1^, which is less than one count every 20 laser pulses. The calculated power per comb incident on the detector is 1.5 × 10^−12^ W, 10^8^ times weaker than commonly used in dual-comb spectroscopy. The average power per comb line is estimated to be 1.2 × 10^−15^ W. The signal is captured by a photon-counting detection module. The second output of the beam splitter produces the trigger signal; the dual-comb interference is detected by a silicon photodiode. The fringe occurring at zero delay acts as a trigger of the multiscaler, which adds up the subsequent scans.

**Fig. 5:**
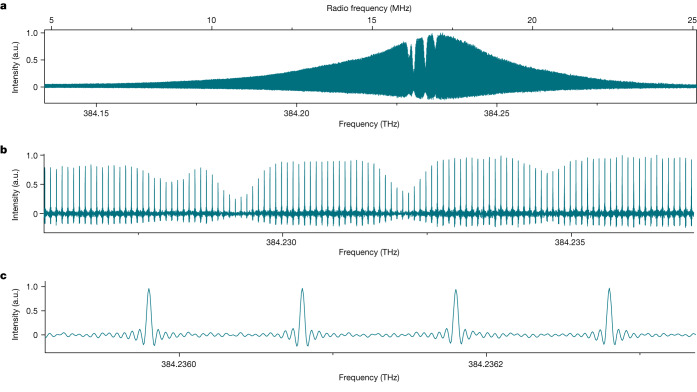
Photon-counting visible dual-comb experimental spectrum with fibre lasers at an average rate of 8.4 × 10^6^ counts s^−1^. The average power per comb line is 1.2 × 10^−15^ W. **a**, Dual-comb spectrum with 1,200 comb lines. **b**, Magnified portion of **a** showing the 5S_1/2_–5P_3/2_ transition of rubidium sampled by the comb lines. **c**, Magnified view of four comb lines. The comb lines show the expected sinc-function instrumental line shape. The full-width at half-maximum of the comb lines is 365 Hz in the radio-frequency domain, corresponding to the Fourier transform limit.

**Extended Data Fig. 7:**
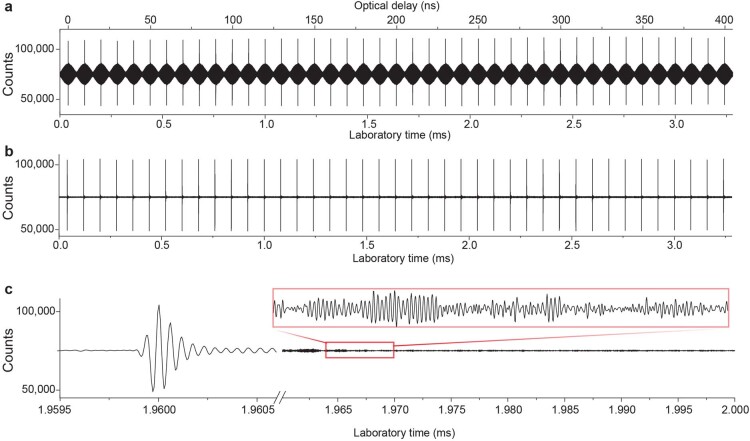
Photon-counting dual-comb interferogram with fiber-laser systems at 384 THz. The time-domain trace results from 1.4 × 10^6^ scans, each with a duration of 3.28 ms. The total measurement time is 76.5 min at a counting rate of 8.4 × 10^6^ counts s^−1^. **a,** Experimentally acquired interferogram of the counting statistics, where the 100-MHz laser pulse trains are partly resolved. **b,** The interferogram of **a** is numerical filtered out with a 40-MHz low-pass filter. **c,** Magnified view of a portion of the dual-comb interferogram in **b** showing the interferometric modulation due to the rubidium transitions.

**Extended Data Fig. 8:**
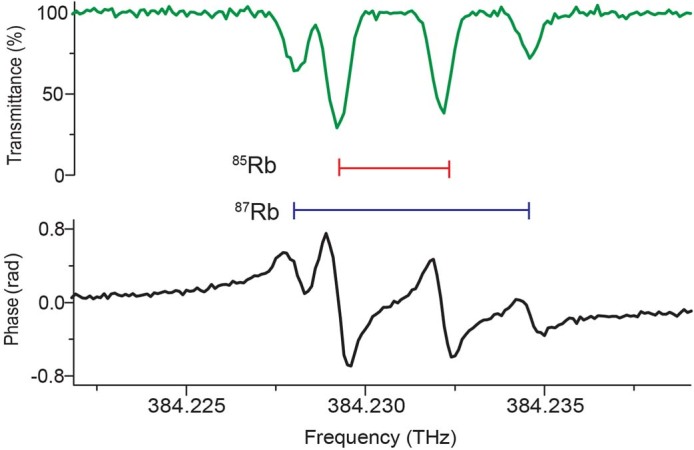
Photon-counting dual-comb transmittance and phase spectra in the region the 5S_1/2_-5P_3/2_ of Rubidium. The spectrum is sampled by the individual comb lines at a resolution of 100 MHz. The spectrum is measured at an average power of 3 × 10^−12^ W and the signal-to-noise ratio is limited by the quantum noise. For each isotope (^85^Rb or ^87^Rb), the resolved hyperfine splitting of the ground state 5S_1/2_ leads to two transitions per isotope.

The multiscaler directly accumulates the time scans, each comprising 5 × 10^5^ time bins over a duration of 3.28 ms. Over a total duration of 4,592 s, the interference signal gradually builds up in the counting statistics. The final interferogram time trace (Extended Data Fig. 7) consists of 41 identical bursts occurring periodically with a period 1/δ*f*_rep_ of 8 × 10^−5^ s. The Fourier transform of the interferogram reveals a transmittance spectrum with resolved comb lines (Fig. 5). The spectrum spans 0.12 THz with 1,200 comb lines well above the noise level (Fig. 5a). The Doppler-broadened 5S_1/2_–5P_3/2_ transitions in ^85^Rb and ^87^Rb, are sampled by the comb lines of 100 MHz spacing (Fig. 5b). The comb lines show a transform-limited width (Fig. 5c). The average signal-to-noise ratio of the absorption baseline is 67, whereas the calculated quantum-noise limited signal-to-noise ratio over 1,200 comb lines is 69. The phase spectrum is simultaneously retrieved. The absorption and dispersion profiles of Rb transitions, sampled by the comb lines at 100 MHz resolution (resolving power 4 × 10^6^), show a full-width at half-maximum of about 600 MHz (Extended Data Fig. 8).

## Discussion

We implement high-resolution linear-absorption dual-comb spectroscopy in the ultraviolet spectral range. Our experiments use two distinct experimental setups with different types of frequency-comb generator, and they establish that the full capabilities of dual-comb spectroscopy are extended to starved-light conditions, at power levels more than a million-fold weaker than those commonly used in dual-comb spectroscopy. By repeatedly achieving a quantum-noise-limited signal-to-noise ratio, an optimal use of the light available for the experiments is achieved. Our photon-level interferometer accurately reproduces the statistics of photon counting, as shown by the signal-to-noise ratio at the fundamental limit, the instrumental line shape that follows the theoretical expectation and the direct referencing of the frequency scale to a radio-frequency clock. The prospect of harnessing dual-comb spectroscopy at very low light levels may seem counterintuitive^10^. Here we have experimentally realized such a milestone, which will unlock new applications. In this section, we discuss the implications and perspectives of our results.

In recent years, dual-comb spectroscopy has emerged as a powerful technique for precise spectroscopy over broad spectral bandwidths. A technique of Fourier transform spectroscopy, it measures the time-domain interference between two frequency combs of slightly different repetition frequencies and reveals the spectrum through harmonic analysis of this interference pattern. However, as a technique relying on frequency measurements rather than on wavelength determination, it does not suffer from the geometric limitations of state-of-the-art interferential or diffractive spectrometers. It promises an as yet untapped potential for high precision and high accuracy. Whereas dual-comb spectroscopy has been mostly used for linear absorption of Doppler- or collision-broadened transitions of small molecules in the gas phase, action spectroscopy has been successfully demonstrated^22,26,27^, for example, with Doppler-free two-photon excitation^26^. Nevertheless, the technique at present uses intense laser beams of an average power higher than several tens of microwatts at the detector and it interrogates samples of millions of atoms or molecules.

In a broader context, however, measuring spectroscopic signatures at extremely low light levels has become critical in many areas of science and technology. These range from precision spectroscopy, in which few atoms or molecules are observed in controlled conditions in which the systematic effects due to interaction between atoms and between atoms and light field are minimized, to light-damageable strongly scattering bio-medical tissues to environmental sounding of the atmosphere over long distances. Our work opens up the prospect of dual-comb spectroscopy at low light levels in these challenging scenarios, which will leverage the host of specific features of the technique.

An exciting forthcoming application is certainly the development of dual-comb spectroscopy in the short-wavelength range, which would unlock precise vacuum- and extreme-ultraviolet molecular spectroscopy over broad spectral spans. Now, broadband ultraviolet spectroscopy is limited in resolution and accuracy^7,37,38^, and at short wavelengths, it relies on unique instrumentation at a unique facility^39,40^. Ultraviolet dual-comb spectroscopy is a much sought-after target, but a challenging one for three primary reasons. First, similar to other lasers, frequency-comb sources that emit directly in the ultraviolet region are not readily available. The current approach to generating ultraviolet frequency-comb radiation involves nonlinear frequency conversion through harmonic generation in crystals (or rare gas jets for shorter wavelengths). However, this process leads to low conversion efficiencies and consequently weak powers. Second, during this conversion process, the phase noise of the comb source is multiplied, typically by the harmonic number. Last, dual-comb spectroscopy relies on interferometry. Many interferometric applications require fringes to be defined to within a hundredth of the wavelength. Achieving such a precision in an interferometer operating at short wavelengths is technically challenging and, for dual-comb spectroscopy, it becomes even more demanding due to the adverse effect of phase-noise multiplication resulting from nonlinear frequency conversion.

Efficient solutions for generating low-noise combs in the ultraviolet and for building dual-comb interferometers with long coherence times have been reported in other contexts. Building on these provides a starting point. Conversely, the pressing issue of low-power handling in dual-comb spectroscopy remained unexplored until our work. In ref. ^4^, fluorescence excitation of a rare gas by a single comb line with a power of 10 pW unlocked linear direct frequency-comb spectroscopy at 63 nm. Here, by controlling the mutual coherence of two mode-locked lasers with 1 femtowatt per comb line, we show an optimal build-up of the counting statistics of their interference signal over times exceeding 1 hour. Our present concept at the femtowatt level will allow to extend the pioneering experiment in ref. ^4^ to broadband dual-comb excitation spectroscopy^26^ in the extreme ultraviolet, to measure at even lower powers, and thus at shorter wavelengths, and potentially to acquire linear-absorption broadband spectra. Furthermore, the generation of frequency combs at frequencies higher than 1,600 THz (wavelengths shorter than 180 nm) has relied on cavity-enhanced high-harmonic generation in a rare gas, a complex process^35^ whose use in dual-comb spectroscopy is daunting. Our work opens up alternative strategies: single-pass high-harmonic generation in a rare gas^41^ or the emerging high-harmonic generation in solids^42^ become conceivable options, even at high repetition frequencies.

## Methods

### Near-ultraviolet frequency-comb generation, photon-counting dual-comb interferometer and experimental spectra

A continuous-wave extended-cavity widely tuneable diode laser emits at a centre frequency of 193 THz (1,550 nm) with an average power of 40 mW. The continuous-wave-laser beam is split into two beams. In one beam, the frequency of the continuous-wave-laser is shifted using an acousto-optic modulator^12,20,43,44^, to avoid aliasing in the dual-comb interferograms. Here the frequency shift is *f*_AOM_ = 40.0 MHz. The centre frequency of the near-ultraviolet dual-comb spectrum is therefore mapped at 160 MHz. In addition, we choose *f*_AOM_ as a multiple of δ*f*_rep_, so that the condition δ*f*_ceo_ = 0(modulo δ*f*_rep_) is met and the individual interferograms are strictly periodic waveforms with a period of 1/δ*f*_rep._ Each beam is modulated by an electro-optic amplitude modulator followed by an electro-optic phase modulator. The phase modulator is driven at a voltage of roughly 4.4 *V*_π_ where *V*_π_ is the voltage required to induce a phase change of π. The amplitude modulator gates the linear part of the up- or down-chirp induced by the phase modulator, leading to a flat-top spectral intensity distribution. To drive repetition frequencies at around 500 MHz, roughly 27 comb lines are generated within a 3 dB bandwidth. Each near-infrared beam is amplified up to 400 mW by an erbium-doped fibre amplifier and it is frequency doubled to 384 THz (780 nm) in a 40 mm-long periodically poled lithium-niobate crystal with a conversion efficiency of 4 × 10^−3^ W^−1^ cm^−1^. The residual 192 THz light is filtered out with a dichroic mirror and the 384 THz beam is focused onto a 10 mm-long BIBO crystal. The conversion efficiency in BIBO is 1 × 10^−4^ W^−1^ cm^−1^. Its low value is due to the low input power in quasi continuous-wave conditions. Two near-ultraviolet combs of 100 lines each, with repetition frequencies *f*_rep_ and *f*_rep_ + δ*f*_rep_ respectively, are generated at around 772 THz. In these experiments, we tune the centre frequency of the combs between 770 and 774 THz but the specifications of the involved instrument allow for tunability between 750 and 784 THz. Typically, we chose *f*_rep_ between 200 and 500 MHz and δ*f*_rep_ between 1.6 MHz and 500 kHz. The span is 50 GHz at a line spacing of 500 MHz (Figs. 2 and 3), and 26 GHz at a line spacing of 200 MHz (Extended Data Fig. 5). The maximum detected count rate of each comb is 2.5 × 10^7^ counts s^−1^, corresponding to an average power for each comb beam of up to 5 × 10^−11^ W.

The intensity fluctuations of each of the ultraviolet comb generators show a standard deviation of 1 × 10^−3^ over 350 s. For accurate frequency measurements, the frequency of the continuous-wave laser is measured against a commercial Er-doped-fibre frequency-comb synthesizer referenced to a global-positioning-system disciplined hydrogen maser. The continuous-wave laser can be phase-locked to a comb line, as in the results in Fig. 3 and Extended Data Fig. 3. The in-loop instabilities amount to less than 1 Hz over half an hour, which means that the optical width of the comb line (of the order of 100 kHz) is transferred to the continuous-wave laser. Alternatively, the continuous-wave laser can be free-running with instabilities monitored by the commercial comb synthesizer and its drifts are up to 2 MHz over 200 s. We did not observe any differences in the signal-to-noise ratio of the dual-comb spectra or in the quality of the spectral line shapes between these two configurations.

The beam of one comb passes through a heated caesium-vapour cell. The two near-ultraviolet beams are then superimposed on a beam splitter to form an interferometer. At one of the outputs of the beam splitter, an optical short-wavelength-pass filter further selects the ultraviolet light and the combined beam is detected by a photon-counting detector. The detector is a photomultiplier of a single-electron response width of 600 ps and a quantum efficiency of 25% at optical frequencies around 772 THz. Photon rates of several 10^7^ photons s^−1^ are detected, corresponding to optical powers incident on the detector of 10^−11^ W. The counts are counted as a function of time by a multiscaler. The multiscaler is started with a trigger signal generated by a frequency division of the 10 MHz clock that synchronizes all electronics in the experiment. Typically, for *f*_rep_ = 500 and δ*f*_rep_ = 1.6 MHz, we set the trigger to 200 kHz. At each trigger signal, the multiscaler adds up the counts to those of previous scans over a total duration of 5 μs, with a time resolution of 160 ps. By accumulating photon-count statistics, an interferogram is reconstituted. The interferograms are zero-filled with a factor of 16 (Fig. 2a,b) or eight (Figs. 2c and 3a,c). A complex Fourier transform of the interferogram reveals the amplitude spectrum and the phase spectrum.

In Fig. 2a, the count rate is 2.7 × 10^7^ counts s^−1^. The strong line at about 770.71 THz (at a radio frequency of 80 MHz) is an artefact corresponding to the frequency-doubled frequency shift of the acousto-optic modulator. Part of the beam that is not deflected by the acousto-optic modulator is coupled into the output fibre. It is then frequency doubled in the periodically poled lithium-niobate crystal and detected by the photon counter. This additive spurious signal can be avoided, for example, by using an acousto-optic modulator with a better diffraction efficiency or by filtering out the 384 THz residual light with a filter of a higher optical density.

In the experiments in Fig. 3a, a caesium-vapour cell of a length of 75 mm is used. For the weak 6S_1/2_–8P_1/2_ transition at around 770 THz (Fig. 3a,b), the Cs vapour pressure is 2 × 10^−1^ Pa (cell temperature 392 K), whereas in Extended Data Fig. 4a,b, both combs experience absorption by Cs and the observed absorption depth is stronger, therefore the cell temperature could be lowered to 378 K (vapour pressure 7 × 10^−2^ Pa). For the 6S_1/2_–8P_3/2_ transition at around 773 THz (Fig. 3c,d and Extended Data Fig. 4c,d), the pressure is 2 × 10^−2^ Pa (cell heated to 359 K). If the signal-to-noise ratio in Fig. 3b and Extended Data Fig. 4 is normalized to an accumulation time of 1 s, one obtains a signal-to-noise ratio at 1 s on the order of 20 s^−1/2^.

For measuring the frequency of the positions of the four 6S_1/2_(*F* = 3,4)–8P_1/2_ and 6S_1/2_(*F* = 3,4)–8P_3/2_ transitions in ^133^Cs (Fig. 3), Doppler profiles are adjusted to the experimental transmittance spectra using a nonlinear least-square fit program. Extended Data Fig. 3 shows the observed spectra of Fig. 3, the results of the fit and the difference ‘observed-fitted’. The difference, at the noise level, does not show any specific signatures. The line centres returned by the fits provide the position of the lines. The positions in ten spectra are averaged for each transition and the results are presented in Extended Data Table 2. We estimate the relative frequency uncertainty of 6 × 10^−9^ for the two most intense lines; This uncertainty is dominated by the statistical uncertainty: the width of the profiles is on the order of 1 GHz and their signal-to-noise ratio is less than 100 and this limits how precisely the line positions can be determined. Our 6S_1/2_(*F* = 3,4)–8P_1/2_ positions are in good agreement with that computed from the more precise measurements in ref. ^34^, in which Doppler-free saturation spectroscopy was implemented. Future work will include reducing the line widths and enhancing the signal-to-noise ratio by background-free Doppler-free spectroscopy^26^.

For *f*_rep_ = 200 MHz, we set δ*f*_rep_ = 0.5 MHz and the frequency of the trigger to 100 kHz. The spectrum (Extended Data Fig. 5) spans over 26 GHz and includes more than 130 comb lines. Whereas the spectral line shapes are more densely sampled at 200 MHz line spacing, the signal-to-noise ratio is 260 at an accumulation time of 328 s.

### Visible-range photon-counting dual-comb interferometer and experimental spectra

Two erbium-doped-fibre mode-locked lasers of a repetition frequency of 100 MHz emit at 192 THz (Extended Data Fig. 6). The repetition frequency *f*_rep_ = 100 MHz and the carrier-envelope offset frequency *f*_ceo_ of the first comb laser (called the master comb generator) are stabilized against the radio-frequency signal of a hydrogen maser, using self-referencing with a *f*-2*f* interferometer. The second comb (called slave comb) has a repetition frequency *f*_rep_ + δ*f*_rep_ with δ*f*_rep_ = −12.5 kHz and a carrier-envelope offset frequency *f*_ceo_ + δ*f*_ceo_. It is stabilized against the first comb through feed-forward control of the relative carrier-envelope offset frequency. This scheme allows long coherence times for the interferometer and direct averaging of the time-domain interferograms over more than 1 hour. The feed-forward control scheme, which uses an external acousto-optic modulator, has been described in detail in ref. ^36^. In addition, as one comb is fully referenced to a radio-frequency clock, absolute calibration of the frequency scale is directly achieved.

In our setup, each laser beam is frequency doubled to 384 THz in a 40-mm-long periodically poled lithium-niobate crystal. The span is limited to 120 GHz by the long crystals to reduce the volume of data, and to adapt to the capabilities of our multiscaler. At the output of the periodically poled lithium-niobate crystals, dichroic mirrors filter out the 192 THz radiation. The 384 THz beam of the master comb generator passes through a 3-cm-long cell with rubidium in natural abundance. The cell is heated to 315.5 K (vapour pressure of Rb 3.3 × 10^−4^ Pa). The beam is then combined on a beam splitter with the beam of the second comb generator. One output of the beam splitter is attenuated to an average optical power of 3 × 10^−12^ W. It is detected by a fibre-coupled single-photon-counting module based on an avalanche photodiode. The counting module detects an average rate of 8.4 × 10^6^ counts per second. The detection efficiency of the module is about 70%. The second output of the beam splitter is detected by a fast silicon photodiode and the central interference fringe provides a trigger signal. The counts of the single-photon-counting module are acquired by a multiscaler triggered by the the fast silicon photodiode. The sampling rate of the multiscaler is 156.25 × 10^6^ samples s^−1^. On average, one count is detected every twelfth laser pulse. As many as 1.4 × 10^6^ triggered sequences, each of 3.28 ms, are summed up to provide, from the photon-counting statistics, a time-domain interference signal (Extended Data Fig. 7) accumulated over a total time of 4,592 s. The interferogram comprises 41 individual interferograms that recur at a period of 1/δ*f*_rep_ = 8 × 10^−5^ s for a total of 512,500 samples, at present limited by the multiscaler capabilities.

The raw interferometric signal shows a significant non-interferential part (Extended Data Fig. 7a); owing to dark counts of the detector, stray light, parasitic light leaking through the fibre before the counting module, spectra of the two combs not perfectly overlapping spectrally, the fringe visibility *V* is 36%. The amplitude of the 41 zero optical-delay bursts remains constant, illustrating that the sequences are efficiently averaged over the time of the experiment. The bandwidth of the electronics does not entirely filter out the pulses at the detector, explaining the residual pulse pattern that is maximum around zero optical delay and minimum at the largest optical delay, in the middle of the interferometric sequence. Simple numerical filtering returns the usual interferogram shape (Extended Data Fig. 7b), where the modulation due to the absorbing rubidium is clearly visible even in the region of the largest optical retardations of 5 ns (Extended Data Fig. 7c). The complex Fourier transform of the interferogram (Extended Data Fig. 7b) provides the complex response (amplitude and phase) of the sample. The unapodized amplitude spectrum, interpolated through fourfold zero-filling of the interferogram, shows well-resolved comb lines with the imprint of the Doppler-broadened 5S_1/2_–5P_3/2_ transitions in ^85^Rb and ^87^Rb (Fig. 5). The spectral envelope of the spectrum has a Gaussian shape determined by the phase-matching conditions in the long periodically poled lithium-niobate crystal. The full-width at half-maximum of the spectral envelope is 38.4 GHz. This corresponds to 384 comb lines spaced at 100 MHz. The individual comb lines show the instrumental line shape of the interferometer, a cardinal sine, which is induced by the finite measurement time. Owing to the good signal-to-noise ratio, more than 1,200 comb lines are measurable over the entire spectral span. Sampling the spectra at the comb line positions reveals the transmittance and dispersion spectra of the Rb transitions (Extended Data Fig. 8).

### Derivation of the quantum-noise-limited signal-to-noise ratio in photon-counting mode

We adapt the formalism developed in refs. ^45,46^ to our experimental situation. We consider a dual-comb interferometer, in which only one output of the interferometer is detected by a photon counter. The time-domain interferogram is composed of a sequence of *L* individual interferograms. An individual interferogram spans over laboratory times between −1/(2δ*f*_rep_) and +1/(2δ*f*_rep_), corresponding to optical delays ranging between −1/(2*f*_rep_) and +1/(2*f*_rep_). As explained above, an individual interferogram is acquired over an accumulation time *T*_indiv_, resulting from the addition, for each time bin, of photon counts over many triggered scans to statistically reconstruct the individual interferogram. Assuming that sufficient statistics have been accumulated, by a proper selection of the sampling and comb parameters, all the individual interferograms are expected to be identical, but for the noise.

At zero optical delay (*t* *=* 0), the quantum-noise-limited signal-to-noise ratio in one individual interferogram is given by

$${\left(\frac{S}{N}\right)}_{t=0}=\frac{{n}_{{\rm{interf}}}}{\sqrt{n+{n}_{{\rm{interf}}}}}$$

where *n* is the number of detector counts corresponding to non-interferometrically modulated signal, accumulated for the time bin at zero optical delay over an integration time of 1/*f*_rep_ and *n*_interf_ is the number of counts that contribute to the interferometric signal for the same time bin (that is, the total number of counts minus the number of counts for non-interferometric contributions).

In an ideal interferometer, one would expect ${n}_{{\rm{interf}}}=n$, leading to ${\left(\frac{S}{N}\right)}_{t=0}=\sqrt{\frac{n}{2}}$. Experimentally, however, many factors contribute to deviations in an additive fashion or in a multiplicative one. Additive contributions include residual stray light or the dark counts of the photon counter. Multiplicative contributions can be due to optical misalignment; the beam splitter may not have the optimal reflection and transmission coefficient; the two interfering combs may not be identical: they may show different power, spectral intensity distribution, polarization and so on.

The fringe visibility *V* can conveniently be introduced: $V=\frac{{n}_{{\rm{interf}},\max }-{n}_{{\rm{interf}},\min }}{{n}_{{\rm{interf}},\max }+{n}_{{\rm{interf}},\min }}\,$, with ${n}_{{\rm{interf}},\max }$ and ${n}_{{\rm{interf}},\min }$ being the maxima and minima of the interference counts, respectively. In an ideal interferometer, *V* = 1. In our experiments, the additive noise is negligible.

In such a case, ${n}_{{\rm{interf}},\max }=n+{n}_{{\rm{interf}}}$ and ${n}_{{\rm{interf}},\min }=n-{n}_{{\rm{interf}}}$, thus ${n}_{{\rm{interf}}}=V\,n$.

Consequently, the signal-to-noise ratio at zero optical delay can be written:

$${\left(\frac{S}{N}\right)}_{t=0}=\frac{V}{\sqrt{1+V}}\sqrt{n},$$

The signal-to-noise ratio ${\left(\frac{S}{N}\right)}_{\nu }$ at the frequency *ν* in the spectrum is related to the signal-to-noise ratio ${\left(\frac{S}{N}\right)}_{t=0}$ at zero optical delay in the time-domain interferogram by the expression:

$${\left(\frac{S}{N}\right)}_{\nu }=\,\sqrt{\frac{2}{K}}\,\frac{B(\nu )}{\bar{{B}_{{\rm{e}}}}}\,{\left(\frac{S}{N}\right)}_{t=0}$$

where *B*(*ν*) is the spectral distribution at the frequency $\nu ,\,{\bar{B}}_{{\rm{e}}}$ is the mean value of the spectral function ${B}_{{\rm{e}}}(\nu )=\frac{1}{2}(B(\nu )+B(-\nu ))$, which accounts for the unphysical negative frequencies. The number of time bins *K* in the interferogram is twice the number of spectral elements in the actual spectral distribution that, according to our sampling conditions here, spans from a frequency 0 to a frequency *f*_rep_/2 on the radio-frequency scale.

Under the simplifying hypothesis that the spectrum is made of *M* comb lines, all of equal intensity, one can express the ratio *B*(*ν*) to $\bar{{B}_{{\rm{e}}}}$ at a frequency *ν* corresponding to a comb line position:

$$\frac{B\left(\nu \right)}{\bar{{B}_{{\rm{e}}}}}=\frac{1/M}{1/K}=\frac{K}{M}$$

Consequently,

$${\left(\frac{S}{N}\right)}_{\nu }={\frac{\sqrt{2K}}{M}\left(\frac{S}{N}\right)}_{t=0}=\sqrt{2}\frac{V}{\sqrt{1+V}}\frac{\sqrt{K}}{M}\sqrt{n}$$

On summing up *L* individual interferograms, the quantum-limited signal-to-noise ratio at the comb line positions becomes:

1 $${\left(\frac{S}{N}\right)}_{\nu }=\sqrt{2}\frac{V}{\sqrt{1+V}}\frac{\sqrt{K}}{M}\sqrt{n\,L}$$

Equation (1) can also be written using the detected photon rate *N*_phot_ (in photons s^−1^) in the interferogram:

$$n=\frac{{N}_{{\rm{phot}}}\,{T}_{{\rm{indiv}}}}{K}$$

where *K* is the number of time bins in the individual interferogram and *T*_indiv_ is the accumulation time for one individual interferogram that has optical delays −1/(2*f*_rep_) to +1/(2*f*_rep_) with time bins of 1/*f*_rep_.

Equation (1) becomes

2 $${\left(\frac{S}{N}\right)}_{\nu }=\sqrt{2}\frac{V}{\sqrt{1+V}}\frac{1}{M}\sqrt{{N}_{{\rm{phot}}}\,{T}_{{\rm{indiv}}}\,L}$$

Moreover, the measurement of *N*_phot_ in the interferogram enables to infer the average power *P* incident on the photon counter.

$${N}_{{\rm{p}}{\rm{h}}{\rm{o}}{\rm{t}}}=\frac{P\,{\rm{Q}}{\rm{E}}\,}{h\nu }$$

where *P* is the average optical power incident on the counter and QE is the counter quantum efficiency.

One can also write the quantum-limited signal-to-noise ratio at the optical frequency *ν* of a comb line as an equation involving the average power rather than the photon counts:

$${\left(\frac{S}{N}\right)}_{\nu }=\frac{\sqrt{2}}{M}\frac{V}{\sqrt{1+V}}\sqrt{\frac{P\,{\rm{Q}}{\rm{E}}\,}{h\nu \,}\,{T}_{{\rm{i}}{\rm{n}}{\rm{d}}{\rm{i}}{\rm{v}}}\,L}=\frac{\sqrt{2}}{M}\frac{V}{\sqrt{1+V}}\sqrt{\frac{P\,{\rm{Q}}{\rm{E}}\,}{h\nu \,}\,{T}_{{\rm{t}}{\rm{o}}{\rm{t}}}\,}$$

where ${T}_{{\rm{tot}}}={T}_{{\rm{indiv}}}\,L$ is the total accumulation time in the entire recording.

## Online content

Any methods, additional references, Nature Portfolio reporting summaries, source data, extended data, supplementary information, acknowledgements, peer review information; details of author contributions and competing interests; and statements of data and code availability are available at 10.1038/s41586-024-07094-9.

### Supplementary information

Supplementary Video 1 **Build-up of the photon statistics in photon-counting dual-comb spectroscopy.** A video illustration of photon-counting dual-comb spectroscopy in the near-ultraviolet spectral region, which shows the build-up of the photon statistics of the interferogram as a function of the accumulation time. As the interferometric time-domain signal builds up, its Fourier transform reveals the dual-comb spectrum with an increasing signal-to-noise ratio. At the end of the recording, the spectrum resembles a dual-comb spectrum at high-power level, and carries the information about the two comb generators with their resolved of comb lines and, when present, about the absorbing sample.

## Figures and Tables

**Extended Data Table 1:**
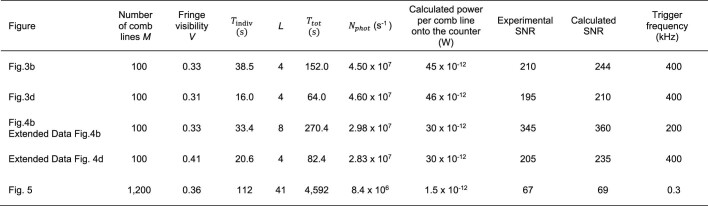
Experimental parameters and calculated quantum-noise limited signal to noise ratio

*L* is the number of individual interferograms in the *T*_indiv_ time sequence. *T*_tot_ = *T*_indiv_*L* is the total accumulation time in the entire recording. *N*_phot_ is the detected photon rate. In all experiments but that of Fig. 5, *f*_rep_ = 500 MHz, δ*f*_rep_ = 1.6 MHz. In Fig. 5, *f*_rep_ = 100 MHz, δ*f*_rep_ = −12.5 kHz. The sampling rate *f*_rep_ and the integration time per time bin is 1/*f*_rep_. The experimental signal-to-noise ratio (SNR) is the inverse standard deviation of the normalized absorption baseline of the amplitude spectrum. The calculated SNR is calculated using Eq. 2. We attribute the slight mismatch between the measured and calculated by the rudimentary model that we use, which neglects additive noise sources and assumes that all comb lines have the same intensity. The calculated average power per comb is half the total power incident on the counter. The photon-counter efficiency is 25% in Figs. 3, 4, Extended Data Fig. 4 and 70% in Fig. 5.

**Extended Data Table 2:**
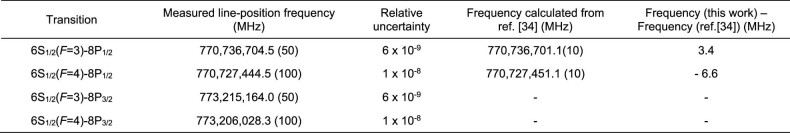
Absolute Frequency measurements of the ^133^Cs 6S_1/2_-8P_1/2_ and 6S_1/2_-8P_3/2_ Doppler-broadened transitions

For comparison, frequencies for the blended 6S_1/2_(*F* = 3)-8P_1/2_ and 6S_1/2_(*F* = 4)-8P_1/2_ have been computed from the Doppler-free measurements in ref. ^34^ where the hyperfine splitting in the upper levels was resolved. We used as relative weights 3/12, 9/12 for the 6S_1/2_(*F* = 3)-8P_1/2_ (*F* = 3,4) lines, respectively and 7/12, 5/12 for the 6S_1/2_(*F* = 4)-8P_1/2_ (*F* = 3,4) resonances, respectively^47^. The numbers in parenthesis are the uncertainties in units of the last digits.

## Data Availability

The data used to produce the plots within this article are available at Edmond, the Open Access Max-Planck Research Data Repository, at 10.17617/3.YAX5OU.
